# Regional Cerebral Associations Between Psychometric Tests and Imaging Biomarkers in Alzheimer’s Disease

**DOI:** 10.3389/fpsyt.2020.00793

**Published:** 2020-08-13

**Authors:** Dennis M. Hedderich, René Drost, Oliver Goldhardt, Marion Ortner, Felix Müller-Sarnowski, Janine Diehl-Schmid, Claus Zimmer, Hans Förstl, Igor Yakushev, Thomas Jahn, Timo Grimmer

**Affiliations:** ^1^Department of Neuroradiology, Klinikum rechts der Isar, School of Medicine, Technical University of Munich, Munich, Germany; ^2^TUM-NIC Neuroimaging Center, Klinikum rechts der Isar, School of Medicine, Technical University of Munich, Munich, Germany; ^3^Department of Psychiatry and Psychotherapy, Klinikum rechts der Isar, School of Medicine, Technical University of Munich, Munich, Germany; ^4^Department of Nuclear Medicine, Klinikum rechts der Isar, School of Medicine, Technical University of Munich, Munich, Germany

**Keywords:** Alzheimer’s disease, magnetic resonance imaging, positron-emission-tomography, biomarkers, cognitive function

## Abstract

Recently, imaging biomarkers have gained importance for the characterization of patients with Alzheimer’s disease; however, the relationship between regional biomarker expression and cognitive function remains unclear. In our study, we investigated associations between scores on CERAD neuropsychological assessment battery (CERAD-NAB) subtests with regional glucose metabolism, cortical thickness and amyloid deposition in patients with early Alzheimer’s disease (AD) using [18F]-fluorodeoxyglucose (FDG), structural MRI, and 11C-Pittsburgh Compound B (PiB) positron emission tomography (PET), respectively. A total of 76 patients (mean age 68.4 ± 8.5 years, 57.9% male) with early AD (median global clinical dementia rating (CDR) score = 0.5, range: 0.5–2.0) were studied. Associations were investigated by correlation and multiple regression analyses. Scores on cognitive subtests were most closely predicted by regional glucose metabolism with explained variance up to a corrected R² of 0.518, followed by cortical thickness and amyloid deposition. Prediction of cognitive subtest performance was increased up to a corrected R² of 0.622 for Word List—Delayed Recall, when biomarker information from multiple regions and multiple modalities were included. For verbal, visuoconstructive and mnestic domains the closest associations with FDG-PET imaging were found in the left lateral temporal lobe, right parietal lobe, and posterior cingulate cortex, respectively. Decreased cortical thickness in parietal regions was most predictive of impaired subtest performance. Remarkably, cerebral amyloid deposition significantly predicted cognitive function in about half of the subtests but with smaller extent of variance explained (corrected R² ≤ 0.220). We conclude that brain metabolism and atrophy affect cognitive performance in a regionally distinct way. Significant predictions of cognitive function by PiB-PET in half of CERAD-NAB subtests suggest functional relevance even in symptomatic patients with AD, challenging the concept of plateauing cortical amyloid deposition early in the disease course. Our results underscore the complex spatial relationship between different imaging biomarkers.

## Introduction

Alzheimer’s disease (AD) is the most common cause for dementia and its prevalence continues to rise in ageing societies (1). Histologically, AD is characterized by pathological β-amyloid plaques and neurofibrillary tau deposits (2–4). *In vivo* characterization of corresponding imaging biomarkers have been strengthened in a currently published research framework (5).

Another hallmark of AD is decline in different cognitive domains, which is typically assessed by standardized neuropsychological testing (6). One of the most widely used procedures is the neuropsychological assessment battery (NAB) of the Consortium to Establish a Registry for Alzheimer’s Disease (CERAD) (7). This neuropsychological assessment covers both general cognitive ability—as determined by the short tests incorporated in the Mini-Mental State examination (MMSE)—and certain cognitive domains such as verbal and non-verbal episodic memory, visuoconstructive capacities, semantic fluency, and executive functions (6, 8).

Imaging techniques are able to provide valuable biomarkers for diagnosis and staging of AD. These are localized or generalized cortical amyloid deposition on Pittsburgh Compound B positron emission tomography (PiB-PET), characteristic glucose hypometabolism on [18F]-fluorodeoxyglucose-PET or cortical thinning derived from magnetic resonance imaging (MRI) (5). In previous studies, the associations between single biomarkers and measures of cognitive decline have been investigated in patients across the spectrum of AD either globally or locally (9–11). Since imaging biomarkers represent distinct aspects of AD and evolve differently during the course of disease, it makes sense to study the three imaging biomarkers amyloid deposition, glucose metabolism, and cortical thickness together (12). However, the relationship between these biomarkers and cognitive function in a single cohort of early AD patients remains unknown.

The present study aims to fill this knowledge gap by examining the regional associations of these three cerebral imaging biomarkers with age-adjusted cognitive function in the same, well-characterized, and relatively large cohort of early AD patients, i.e. patients with prodromal and mild stages of AD (13), using a three-step approach: First, correlation analyses were performed in order to get an overview of the relationship between cortical biomarkers and cognitive function. In a second step, we aimed at identifying the single most predictive cortical brain region for each cognitive subtest performance. Third, we examined which set of cortical brain regions led to the highest predictive power regarding different aspects of cognitive function for the three imaging biomarkers both separately and together. We hypothesized that associations would be closest for glucose metabolism and loosest for amyloid deposition. Furthermore, we hypothesized an increase in predictive power for regression models with biomarker information from multiple ROIs and multiple modalities.

## Materials and Methods

### Participants

All participants were referred to the Center for Cognitive Disorders (Department of Psychiatry and Psychotherapy, Klinikum rechts der Isar, Technical University of Munich) for the evaluation of a cognitive disorder and a possibly underlying neurodegenerative disease. Inclusion criteria were: Fulfillment of National Institute on Aging-Alzheimer’s Association (NIA-AA) criteria for probable Alzheimer’s disease dementia (14), very mild to moderate clinical dementia severity, and characteristic findings on FDG-PET (hypometabolism of the temporoparietal junction and the posterior cingulate cortex with relative sparing of the primary somatosensory and somatomotor cortices) (15). Exclusion criteria were: (1) fulfillment of diagnostic criteria for dementia with proven underlying non-AD pathology (e.g. Normal Pressure Hydrocephalus, presence of vascular dementia according to the NINDS-AIREN criteria) (16), (2) pathological findings on MRI such as advanced leukoencephalopathy, strategic infarctions, intracranial aneurysms, or arteriovenous malformations, or (3) possible alternative causes for neurocognitive impairment such as antidepressant or antipsychotic medication, derangement of blood electrolytes, or drug abuse. Amyloid imaging by [^11^C] PiB PET was used as a research add-on.

All patients provided written informed consent regarding the scientific evaluation of their data. The study protocol was approved by the German radiation protection authorities and the ethics committee of the School of Medicine of the Technical University of Munich, Munich, Germany (reference number 1285/05).

### Clinical and Cognitive Assessment

All tests were performed by trained experts, neuropsychological testing and brain MRI were performed within 60 days for every participant. Clinical Dementia Rating scale (CDR) global score served to clinically grade the severity of dementia (0 = no impairment, 0.5 = very mild dementia, 1 = mild dementia, 2 = moderate dementia, 3 = severe dementia) and the sum of subscores (CDR SOB) indicating the grade of impairment in six categories (memory, orientation, judgment and problem solving, community affairs, home and hobbies, personal care) were calculated (8, 17). Mini-Mental State examination (MMSE) was used to capture global cognitive deficits (18). All participants underwent neuropsychological testing using the full neuropsychological assessment battery by the Consortium to Establish a Registry for Alzheimer’s Disease (CERAD-NAB) (7). Raw values of CERAD-NAB subtests of study participants were transformed to z-scores adjusting for age, sex, and years of education using CERAD-Plus 1.0 for Microsoft Excel (available at: https://www.memoryclinic.ch/de/main-navigation/neuropsychologen/cerad-plus/auswertungprogramme/cerad-plus-10-excel/). Normative values within this software package were derived from a reference cohort consisting of 617 healthy control participants between 53 and 92 years of age as described by Berres et al. (19).

### MRI Data Acquisition and Analysis

All patients underwent structural magnetic resonance imaging on a 1.5 Tesla Siemens Magnetom Symphony platform (Siemens, Erlangen, Germany) at the time of initial presentation in order to exclude major structural abnormalities and to evaluate atrophy. The imaging protocol comprised a three-dimensional, T1-weighted, gradient echo sequence that was used for further analyses. Imaging parameters were as follows: TR = 1520 ms, TE = 3.93 ms, matrix size = 256 x 256, flip angle = 15°, slice thickness = 1 mm. In addition to visual assessment, scans were normalized to a MNI template using SPM 8, warping parameters were recorded for later normalization of individual FDG-PET and PiB-PET images as previously described (20, 21). Cortical thickness was calculated following the established–reconall pipeline in Freesurfer (Version 5.1.0) (22, 23). Cortical segmentation was checked visually and deemed satisfactory in all cases. Mean cortical thickness values were extracted for 31 cortical regions-of-interest per hemisphere as defined in the Desikan-Killiany-Tourville (DKT) protocol (24). Additionally, a global cortical thickness score per participant was calculated using the following formula: (Mean_cortical_thickness [ROI1] x Surface_Area [ROI1] + Mean_cortical_thickness [ROI2] x Surface_Area [ROI2] + … + Mean_cortical_thickness [ROI62] x Surface_Area [ROI62])/(Surface_Area [ROI1] + Surface_Area [ROI2] + … + Surface_Area [ROI62]).

### PET Data Acquisition

Imaging studies (MRI, FDG-PET, and PiB-PET) were performed within 30 days according to the study protocol. All participants were imaged under standard resting condition (eyes closed in dimmed ambient light) using a Siemens ECAT HR+ PET scanner (CTI, Knoxville, TN, USA) (25). Participants were positioned with the head parallel to the canthomeatal line within the gantry. Image data were acquired in 3D mode with a total axial field of view of 15.5 cm. A transmission scan was acquired after completion of the emission scan for attenuation correction. A 3-dimensional attenuation-weighted ordered-subsets expectation maximization iterative reconstruction algorithm (AW OSEM 3D) was applied with four iterations and eight subsets, Gaussian smoothing of 10 mm in full width at half maximum, and a zoom of 1.

PET imaging was started 30 min after injection of about 185 MBq [^18^F] FDG. A sequence of one frame of 10 min and two frames of 5 min was started and later summed into a single frame. Primarily, an experienced observer for quality control and individual assessment performed visual analysis of all FDG-scans.

For amyloid imaging, patients were injected with about 370 MBq [^11^C] PiB at rest. Thirty minutes later, patients were placed in the scanner and at 40 min post-injection, three 10-min frames of data acquisition were started and later summed into a single frame (40–70 min).

### PET Data Analysis

[^18^F] FDG and [^11^C] PiB PET scans were analyzed using SPM 8 (http://www.fil.ion.ucl.ac.uk/spm/) running on MATLAB (Version 12, The MathWorks Inc., Natick, Massachusetts, United States). PET analyses were performed following standard procedures as published previously (26–28). Images were realigned using a least squares approach and a six parameter (rigid body) spatial transformation to account for minimal motion artifacts and spatially normalized to MNI space using the warping parameters from the individual normalization of structural MRI scans. Furthermore, images were smoothed with a 10 mm x 10 mm x 10 mm full width at half maximum (FWHM) Gaussian kernel. After normalization to MNI space, PET imaging data was parcellated to ROIs based on the DKT atlas (24) using the free software tool AMIDE (29). Signal intensities of [^18^F] FDG and [^11^C] PiB imaging data were normalized to the pons and the vermis cerebelli, respectively and reported as standardized uptake value ratios (SUVR). In addition to ROI-based analyses, a mean value of global grey matter signal intensity per each individual was calculated.

### Statistical Analysis

Mean values of ROI-based cortical thickness and relative signal intensities of FDG-PET and PiB-PET images were extracted for external analyses in IBM SPSS (Version 23 IBM Corp.) Mean values, standard deviation, coefficient of variation, minimum, and maximum values were calculated for demographic and test variables. In order to explore correlations of regional imaging data with neuropsychiatric test results, Pearson correlation analyses were performed to identify the regional pattern of correlation with z-scores of cognitive tests adjusted for sex, age, and years of education. In addition we performed multiple linear regression analyses in order to identify a) the most predictive region and b) the most predictive set of regions associated with cognitive z-scores adjusted for sex, age, and years of education. For multiple linear regression analyses, all 62 brain regions were initially entered followed by stepwise selection of significant variables (in p<0.05, out p >0.10). In order to account for influences of age and disease severity on the three biomarkers, we added age and CDR-SOB as covariates into the model resulting from the stepwise regression approach described above. The alpha level was set at α = 0.05. The Bonferroni method was used as correction for multiple comparisons (Pearson correlation: 62 brain regions, multiple linear regression analyses: three biomarkers).

## Results

### Sample Characteristics

#### Clinical and Demographic Information

A total of 76 patients (mean age 68.4 ± 8.5 years, range 50–83 years, 57.9% male) with early AD were included in this study. Mean time of education was 12.6 years ± 2.4 years. Median CDR global was 0.5, range: 0.5–2.0 and median CDR sum of boxes was 3.0, range: 0.5–11.0). Visual reading of PiB-PET showed positive cortical amyloid deposition in all cases. Mean z-scores of CERAD-NAB subtests, adjusted to sex, age, and time of education are given in **Table 1**. In cases of CERAD-NAB subtests with n < 76 participants, the individuals refused to complete the test and the result could not be evaluated.

**Table 1:** Z-scores of CERAD-NAB subtests.

	n	Mean	SD	Min	Max
**Verbal Fluency**	75	-1.64	1.01	-3.66	0.29
**Mod. Boston Naming Test**	75	-1.24	1.47	-4.49	1.35
**MMSE**	76	-2.99	1.72	-7.21	0.78
**Word List—Immediate Recall**	75	-2.56	1.48	-6.45	0.92
**Word List—Delayed Recall**	75	-2.30	1.27	-4.64	0.88
**Word List—Savings**	74	-2.22	1.92	-5.31	3.52
**World List—Discriminability**	71	-1.74	1.61	-5.98	1.28
**Constructional Praxis**	76	-1.04	1.88	-7.65	1.74
**Figures—Recall**	76	-2.39	1.48	-5.56	1.73
**Figures—Savings**	75	-1.88	1.26	-3.78	2.60

CERAD-NAB, Consortium to Establish a Registry for Alzheimer’s Disease—Neuropsychological Assessment Battery; MMSE, Mini-Mental State Examination; SD, standard deviation; Min, Minimum; Max, Maximum. n indicates number of participants for particular subtest.

#### Variance of Cerebral Biomarkers

In order to describe the variance and dynamic range of cerebral biomarkers, we calculated global and regional means presented as SUVRs, together with standard deviations and coefficients of variation. On a global level, amyloid deposition showed the highest variance (SUVR 1.727 ± 0.336 [a.u.], coefficient of variation: 19.4%), followed by glucose metabolism (SUVR 1.395 ± 0.179 [a.u.], coefficient of variation: 12.8%) and cortical thickness (mean 2.24 ± 0.26 mm, coefficient of variation: 11.5%). ROI-based coefficients of variation ranged from 15.1%–23.9%, 8.2%–17.8%, and 8.9%–20.9% for PiB uptake, FDG uptake, and cortical thickness, respectively. Detailed ROI-based characteristics are given in supplementary **Tables S1**-**S3**.

### Correlation Analyses

#### Correlation of Global Imaging Data With Neuropsychological Test Scores

Global normalized FDG uptake correlated significantly with three CERAD-NAB subtests, explicitly with the MMSE (r = 0.419, p = 0.002), Figures—Recall (r = 0.412, p = 0.002), and Figures—Savings (r = 0.360, p = 0.017). No significant correlations of neuropsychological test scores with global cortical thickness and global, normalized PiB-PET signal intensity were observed.

#### Correlation of Neuropsychological Test Scores With Regional Amyloid Deposition

**Figure 1:**
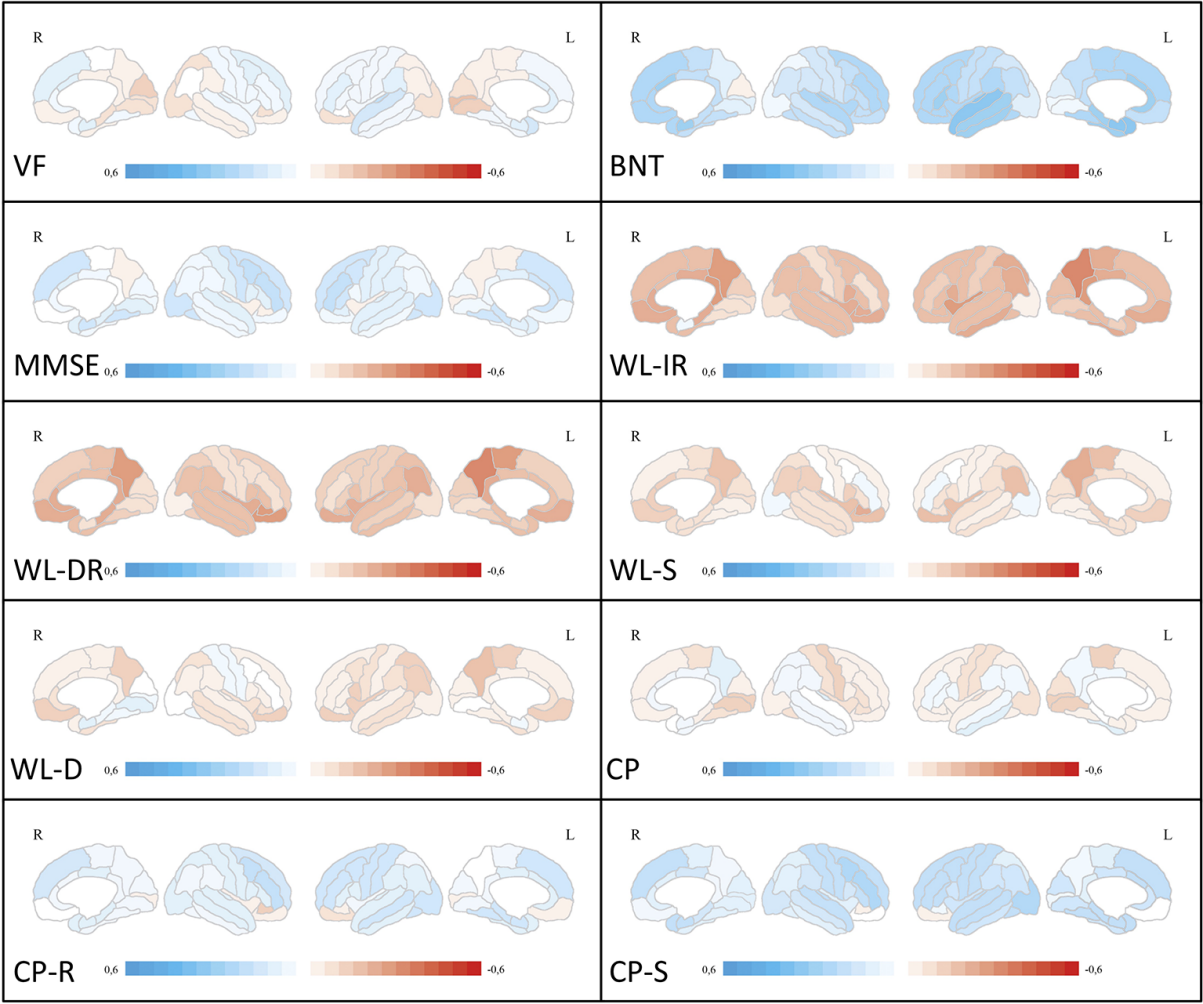
Correlation analyses between ROI-based amyloid deposition and neuropsychological test scores. Medial and lateral projections of the right and left hemisphere are shown. Red color depicts negative correlations (Pearson’s r), blue color depicts positive correlations. Maximum Pearson’s r is set at -0.6 and 0.6, respectively. Verbal fluency (VF), Boston Naming Test (BNT), Mini-Mental State examination (MMSE), Word List Immediate Recall (WL-IR), Word List Delayed Recall (WL-DR), Word List Savings (WL-S), Word List Discriminability (WL-D), Constructional Practice (CP), Figures Recall (CP-R), Figures Savings (CP-S). ROI, region of interest.

After correction for multiple comparisons, no subtest z-score showed a significant correlation with ROI-based amyloid deposition as measured by [^11^C] PiB PET. Pearson’s r for the correlation between ROI-based amyloid deposition and neuropsychological test scores is visualized in **Figure 1**.

#### Correlation of Neuropsychological Test Scores With Regional Glucose Metabolism

**Figure 2:**
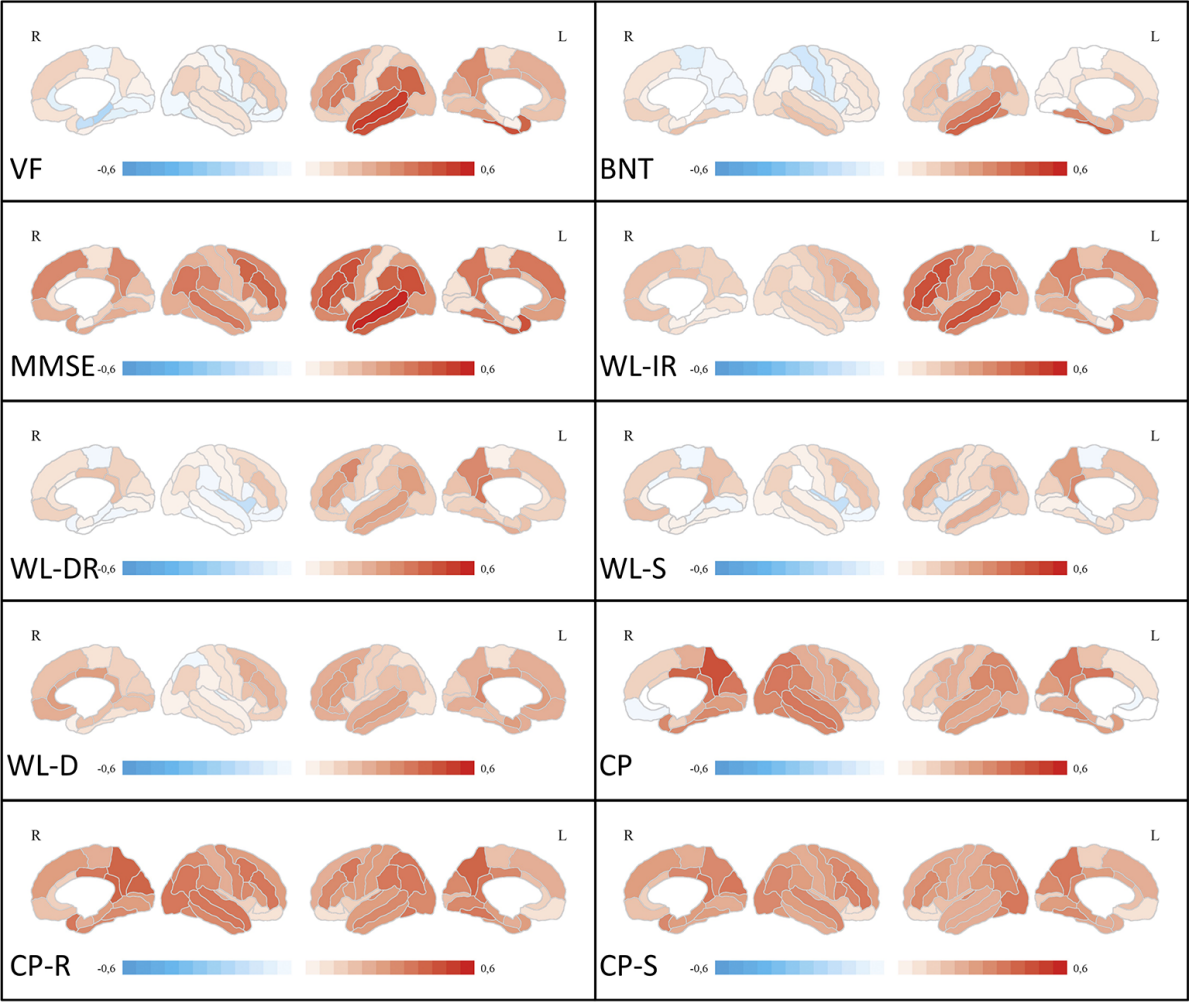
Correlation analyses between ROI-based FDG uptake and neuropsychological test scores. Medial and lateral projections of the right and left hemisphere are shown. Red color depicts positive correlations (Pearson’s r), blue color depicts negative correlations. Maximum Pearson’s r is set at -0.6 and 0.6, respectively. Verbal fluency (VF), Boston Naming Test (BNT), Mini-Mental State examination (MMSE), Word List Immediate Recall (WL-IR), Word List Delayed Recall (WL-DR), Word List Savings (WL-S), Word List Discriminability (WL-D), Constructional Practice (CP), Figures Recall (CP-R), Figures Savings (CP-S). FDG, [18F]-fluorodeoxyglucose; ROI, region of interest.

After correction for multiple comparisons for 62 brain regions, no significant correlations were found between Word List—Delayed Recall, Word List—Savings, and Word List—Discriminability and regional glucose metabolism. Most significant correlations were found for MMSE, with a predominance of left-sided frontotemporal regions. For a graphical overview of Pearson’s r coefficients, please see **Figure 2**. Cognitive tasks demanding verbal capacities correlated mostly with left-sided temporal regions. Cognitive tasks including constructional praxis and visuospatial coordination correlated predominantly with right hemispheric, parietal ROIs. For a graphical overview about correlations, irrespective of statistical thresholds between ROIs and z-scores of cognitive tasks, please see **Figure 2**. Detailed correlation coefficients for significant ROIs after Bonferroni correction are given in **Table S4**.

#### Correlation of Neuropsychological Test Scores With Regional Cortical Thickness

**Figure 3:**
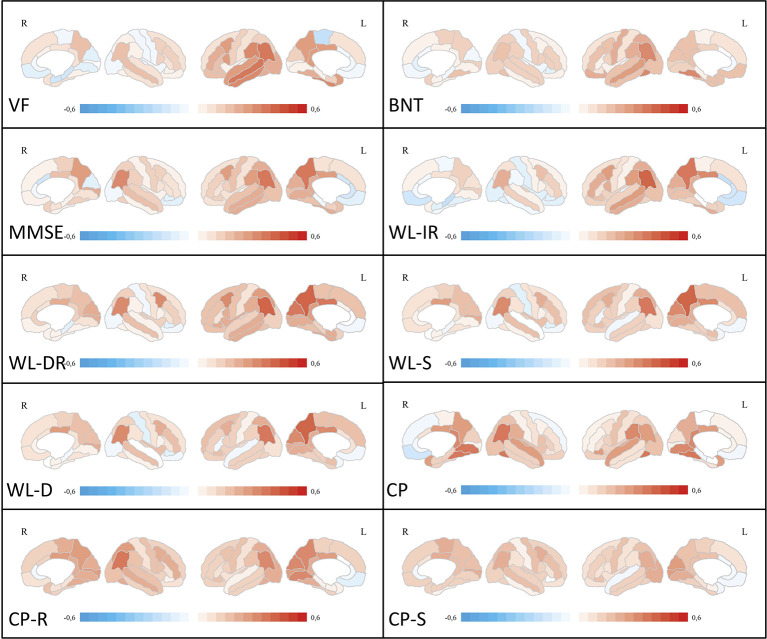
Correlation analyses between ROI-based cortical thickness and neuropsychological test scores. Medial and lateral projections of the right and left hemisphere are shown. Red color depicts positive correlations (Pearson’s r), blue color depicts negative correlations. Maximum Pearson’s r is set at -0.6 and 0.6, respectively. Verbal fluency (VF), Boston Naming Test (BNT), Mini-Mental State examination (MMSE), Word List Immediate Recall (WL-IR), Word List Delayed Recall (WL-DR), Word List Savings (WL-S), Word List Discriminability (WL-D), Constructional Practice (CP), Figures Recall (CP-R), Figures Savings (CP-S). ROI, region of interest.

After correction for multiple comparisons for 62 brain regions, no significant correlations were found between Modified Boston Naming Test, Word List—Discriminability, Figures—Recall and Figures Savings and regional cortical thickness. In general, fewer significant correlations with cognitive z-scores were seen for cortical thickness than for glucose metabolism. Most significant correlations overall were found for the parietal lobe on the left and right side. The left-sided inferior parietal lobule correlated with Word List task performance. The fusiform gyrus on the left and right side correlated with constructional praxis tasks. For a graphical overview of Pearson’s r coefficients, irrespective of statistical thresholds, please see **Figure 3**. Detailed correlation coefficients for significant ROIs after Bonferroni correction are given in **Table S5**.

### Multiple Linear Regression Analyses

#### Prediction of Neuropsychological Performance by Cortical Amyloid Deposition

Detailed results for regional amyloid deposition predicting CERAD-NAB subtest performance in the single most predictive ROI and the most predictive set of ROIs can be found in **Table 2**. Five out of 10 CERAD-NAB subtests could be predicted by amyloid PiB binding in single ROI. Three predictions remained significant after correction for multiple comparisons: Modified Boston Naming Test, Word List—Immediate Recall, and Word List—Delayed Recall. In the significant linear regression analyses, corrected R^2^ ranged from 0.081–0.108 for the single regions.

**Table 2:** Relationship between cognitive performance and ROI-based amyloid deposition.

CERAD-NAB subtest	Single ROI	β	T	p*	F	Corr. R^2^		Set of ROIs	β	T	p*	F	Corr. R^2^	p
**Verbal Fluency**	*No significant ROI*							*No significant ROI*						
**Mod. BNT**	Left entorhinal cortex	0.346	3.171	0.006	10.057	0.108		Left entorhinal cortex	0.346	3.171	0.006	10.057	0.108	0.002
**MMSE**	*No significant ROI*							*No significant ROI*						
**Word List—Immediate Recall**	Left precuneus	-0.305	-2.755	0.021	7.587	0.081		Left precuneus	-0.683	-3,747	0.001	7.352	0.145	0.001
								Right fusiform gyrus	0.466	2.559	0.039			
**Word List—Delayed Recall**	Left precuneus	-0.322	-2.927	0.015	8.567	0.092		Left precuneus	-0.788	-4.01	<0.001	8.623	0.169	<0.001
								Left caudal middle frontal gyrus	0.551	2.808	0.018			
**Word List—Savings**	Right inferior frontal gyrus, pars orbitalis	-0.241	-2.14	0.108	4.581	0.046		Right inferior frontal gyrus, pars orbitalis	-0.322	-2.14	0.108	4.581	0.046	0.036
**World List—Discriminability**	*No significant ROI*							*No significant ROI*						
**Constructional Praxis**	*No significant ROI*							*No significant ROI*						
**Figures—Recall**	*No significant ROI*							*No significant ROI*						
**Figures—Savings**	Right caudal middle frontal gyrus	0.246	2.182	0.096	4.762	0.048		Right caudal middle frontal gyrus	0.801	4.154	<0.001	8.060	0.220	<0.001
								Right lateral orbitofrontal gyrus	-0.904	-4.273	<0.001			
								Left parahippocampal gyrus	0.318	2.115	0.114			

Relationship between regional amyloid deposition as measured by [^11^C] PiB-PET and cognitive performance as measured by z-scores on CERAD-NAB subtests. Coefficients of determination are given for the single most predictive ROI and a set of most predictive ROIs with regard to CERAD-NAB subtests performance. P-values are Bonferroni corrected (*) for testing three different biomarkers.

β, standardized regression coefficient; CERAD-NAB, Consortium to Establish a Registry for Alzheimer’s Disease—Neuropsychological Assessment Battery; Corr., corrected; MMSE, Mini-Mental State Examination; Mod. BNT, Modified Boston Naming Test; PET, Positron emission tomography; PIB, Pittsburgh Compound B; ROI, region of interest.

Five out of 10 CERAD-NAB subtests could be predicted by regional amyloid PiB binding in a set of ROIs, namely Modified Boston Naming Test, Word List—Immediate Recall, Word List—Delayed Recall, Word List—Savings and Figures—Savings with corrected R² ranging between 0.108–0.220.

Interestingly, regional amyloid deposition predicted CERAD-NAB subtest performance showing both positive and negative β-coefficients and thus both positive and inverse relationships. The five subtests Verbal Fluency, MMSE, Word List—Discriminability, Constructional praxis, and Figures Recall could neither be predicted by a single ROI nor a set of regions.

#### Prediction of Neuropsychological Performance by Regional Glucose Metabolism

Detailed results for cortical FDG uptake predicting CERAD-NAB subtest performance in the single most predictive ROIs and the most predictive set of ROIs can be found in **Table 3**. Regional glucose metabolism was able to significantly predict performance in every CERAD-NAB subtest based on both a single ROI and a set of ROIs. Corrected R^2^ values ranged from 0.083–0.300 for single ROI predictions and from 0.176–0.518 for multiple ROI regression analyses. All β-coefficients were positive in the single ROI analyses and ranged from 0.309–0.556. The single most predictive ROIs were located in the left lateral temporal lobe and the (posterior) cingulate cortex and precuneus. β-coefficients were both positive and negative when using multiple ROIs for the prediction of CERAD-NAB subtest performance.

**Table 3:** Relationship between cognitive performance and ROI-based glucose metabolism.

CERAD-NAB subtest	Single ROI	β	T	p*	F	Corr. R^2^	Set of ROIs	β	T	p*	F	Corr. R^2^	p
**Verbal Fluency**	Left middle temporal gyrus	0.507	5.056	<0.001	25.566	0.247	Right parahippocampal gyrus	-0.473	5.001	<0.001	19.238	0.422	<0.001
							Left transverse temporal gyrus	0.306	2.956	0.012			
							Left inferior temporal gyrus	0.465	4.582	<0.001			
**Mod. BNT**	Left inferior temporal gyrus	0.435	4.152	<0.001	17.241	0.178	Left inferior temporal gyrus	0.466	4.504	<0.001	10.082	0.266	<0.001
							Right postcentral gyrus	-0.555	-3.243	0.006			
							Right superior frontal gyrus	0.383	2.218	0.090			
**MMSE**	Left middle temporal gyrus	0.556	5.762	<0.001	33.195	0.300	Left middle temporal gyrus	0.228	1.751	0.252	14.432	0.518	<0.001
							Left rostral middle frontal gyrus	0.594	3.765	<0.001			
							Left postcentral gyrus	-0.677	-4.946	<0.001			
							Right caudal middle frontal gyrus	0.648	4.070	<0.001			
							Right inferior frontal gyrus pars triangularis	-0.491	-3.139	0.006			
							Left fusiform gyrus	0.297	2.358	0.063			
**Word List—Immediate Recall**	Left middle temporal gyrus	0.476	4.653	<0.001	21.652	0.216	Left middle temporal gyrus	0.290	2.464	0.048	12.699	0.319	<0.001
							Left rostral middle frontal gyrus	0.647	3.630	0.003			
							Right superior frontal gyrus	-0.444	-2.765	0.021			
**Word List—Delayed Recall**	Left isthmus of the cingulate gyrus	0.378	3.51	0.003	12.317	0.131	Left isthmus of cingulate gyrus	0.505	4.641	<0.001	12.134	0.229	<0.001
							Right insula	-0.351	-3.223	0.006			
**Word List—Savings**	Left isthmus of the cingulate gyrus	0.309	2.793	0.021	7.801	0.083	Left isthmus of cingulate gyrus	0.587	5.025	<0.001	8.778	0.384	<0.001
							Right medial orbitofrontal gyrus	0.851	4.870	<0.001			
							Right lateral orbitofrontal gyrus	-0.798	-4.210	<0.001			
							Left insula	-0.426	-3.263	0.006			
							Right lingual gyrus	-0.362	-2.830	0.018			
							Right precentral gyrus	0.282	2.103	0.117			
**World List—Discriminability**	Left isthmus of the cingulate gyrus	0.333	3.04	0.009	9.245	0.099	Left isthmus of the cingulate gyrus	0.403	3.530	0.003	6.883	0.190	<0.001
							Right transverse temporal gyrus	-0.276	-2.435	0.051			
							Left entorhinal cortex	0.234	2.217	0.09			
**Constructional Praxis**	Right precuneus	0.464	4.51	<0.001	20.34	0.205	Right precuneus	0.567	5.206	<0.001	12.466	0.314	<0.001
							Right medial orbitofrontal gyrus	-0.402	-3.494	0.003			
							Left inferior temporal gyrus	0.260	2.414	0.054			
**Figures—Recall**	Right precuneus	0.439	4.205	<0.001	17.68	0.182	Right precuneus	0.439	4.205	<0.001	17.680	0.182	<0.001
**Figures—Savings**	Right isthmus of the cingulate gyrus	0.393	3.673	0.001	13.489	0.143	Right isthmus of the cingulate gyrus	0.293	2.525	0.042	9.006	0.176	<0.001
							Left occipital complex	0.232	1.995	0.15			

Relationship between regional glucose metabolism as measured by [^18^F] FDG-PET and cognitive performance as measured by z-scores on CERAD-NAB subtests. Coefficients of determination are given for the single most predictive ROI and a set of most predictive ROIs with regard to CERAD-NAB subtests performance. P-values are Bonferroni corrected (*) for testing three different biomarkers.

β, standardized regression coefficient; CERAD-NAB, Consortium to Establish a Registry for Alzheimer’s Disease—Neuropsychological Assessment Battery; Corr., corrected; FDG, [18F]-fluorodeoxyglucose; MMSE, Mini-Mental State Examination; PET, Positron emission tomography; ROI, region of interest.

#### Prediction of Neuropsychological Performance by Regional Cortical Thickness

Detailed results for regional cortical thickness predicting CERAD-NAB subtest performance in the single most predictive ROIs and the most predictive set of ROIs can be found in **Table 4**. ROI-based measurement of cortical thickness was able to significantly predict performance in every CERAD-NAB subtest, and in all but the Word List—Discriminability after correction for multiple testing, based on both a single ROI and a set of ROIs. Corrected R^2^ values ranged from 0.065–0.178 for single ROI and from 0.151–0.520 for multiple ROI regression analyses. All β-coefficients were positive in the single ROI analyses and ranged from 0.279–0.434. Single most predictive ROIs were located in the lateral and medial parietal lobe and the inferior temporal lobe. β-coefficients were both positive and negative when using multiple ROIs for the prediction of CERAD-NAB subtest performance.

**Table 4:** Relationship between cognitive performance and ROI-based cortical thickness.

CERAD-NAB subtest	Single ROI	β	T	p*	F	Corr. R^2^	Set of ROIs	β	T	p*	F	Corr. R^2^	p
**Verbal Fluency**	Left inferior parietal lobule	0.377	3.504	<0.001	12.280	0.131	Left inferior parietal lobule	0.335	1.936	0.171	10.907	0.398	<0.001
							Left paracentral gyrus	-0.441	-3.940	<0.001			
							Right lingual gyrus	-0.447	-3.752	<0.001			
							Left superior temporal gyrus	0.316	2.612	0.033			
							Left precuneus	0.371	2.188	0.096			
**Mod. BNT**	Left fusiform gyrus	0.309	2.795	0.021	7.813	0.083	Left fusiform gyrus	0.480	3.849	<0.001	7.677	0.151	0.001
							Right inferior frontal gyrus. pars orbitalis	-0.328	-2.630	0.03			
**MMSE**	Left precuneus	0.395	3.701	0.001	13.697	0.145	Left precuneus	0.706	6.033	<0.001	10.067	0.326	<0.001
							Right cuneus	-0.400	-3.411	0.003			
							Right entorhinal cortex	0.259	2.631	0.03			
							Left rostral anterior cingulate cortex	-0.221	-2.187	0.096			
**Word List—Immediate Recall**	Left inferior parietal lobule	0.413	3.897	<0.001	15.183	0.159	Left parietal inferior lobule	0.598	4.472	<0.001	10.857	0.345	<0.001
							Right supramarginal gyrus	-0.418	-3.234	0.006			
							Left medial orbitofrontal gyrus	-0.284	-2.678	0.027			
							Left posterior cingulate cortex	0.302	2.426	0.054			
**Word List—Delayed Recall**	Left inferior parietal lobule	0.434	4.148	<0.001	17.205	0.178	Left parietal inferior lobule	0.457	3.434	0.003	13.232	0.449	<0.001
							Right supramarginal gyrus	-0.793	-4.868	<0.001			
							Right parietal inferior lobule	0.723	3.626	<0.001			
							Right lateral occipital complex	-0.476	-3.702	<0.001			
							Right cuneus	0.357	2.930	0.015			
**Word List—Savings**	Left precuneus	0.390	3.645	0.001	13.285	0.141	Left precuneus	0.629	5.209	<0.001	13.805	0.255	<0.001
							Right postcentral gyrus	-0.423	-3.507	<0.001			
**World List—Discriminability**	Left posterior cingulate cortex	0.269	2.404	0.057	5.780	0.060	Left posterior cingulate cortex	0.403	3.530	0.057	5.780	0.060	0.019
**Constructional Praxis**	Right fusiform gyrus	0.407	3.834	<0.001	14.701	0.154	Right fusiform gyrus	0.265	2.086	0.123	14.520	0.520	<0.001
							Right medial orbitofrontal gyrus	-0.357	-3.207	0.006			
							Right posterior cingulate cortex	0.310	2.758	0.021			
							Right superior frontal gyrus	-0.551	-4.099	<0.001			
							Left isthmus of the cingulate gyrus	0.375	3.753	<0.001			
							Right lingual gyrus	0.420	3.361	0.003			
**Figures—Recall**	Right inferior parietal lobule	0.366	3.382	0.003	11.435	0.122	Right inferior parietal lobule	0.780	4.011	<0.001	9.322	0.182	<0.001
							Right supramarginal gyrus	-0.491	-2.525	0.042			
**Figures—Savings**	Left cuneus	0.279	2.500	0.045	6.250	0.065	Left cuneus	0.279	2.500	0.045	6.250	0.065	0.015

Relationship between regional cortical thickness as measured by structural MRI and cognitive performance as measured by z-scores on CERAD-NAB subtests. Coefficients of determination are given for the single most predictive ROI and a set of most predictive ROIs with regard to CERAD-NAB subtests performance. P-values are Bonferroni corrected (*) for testing three different biomarkers.

β, standardized regression coefficient; CERAD-NAB, Consortium to Establish a Registry for Alzheimer’s Disease—Neuropsychological Assessment Battery; Corr., corrected; MMSE, Mini-Mental State Examination; MRI, Magnetic Resonance Imaging; ROI, region of interest.

#### Prediction of Neuropsychological Performance by Multimodal Biomarker Information

In order to investigate the interplay of the three cerebral imaging biomarkers, we included cortical thickness, amyloid deposition, and glucose metabolism into the same regression model. Interestingly, we observe that for all cognitive tests, biomarkers of different entities from distinct regions are included. Additionally, the variance explained by the multimodal regression model mostly increases substantially compared to unimodal regression models. Detailed results are given in **Table 5**.

**Table 5:** Relationship between cognitive performance and multimodal ROI-based biomarker information.

CERAD-NAB subtest	Biomarker	ROI	β	T	p	F	Corr. R^2^	p
**Verbal Fluency**	FDG	Left middle temporal gyrus	0.633	6.864	<0.001	14.516	0.422	<0.001
	FDG	Right parahippocampal gyrus	-0.341	-3.773	<0.001			
	CTh	Right inferior frontal gyrus, pars triangularis	0.414	3.291	0.002			
	CTh	Left superior frontal gyrus	-0.287	-2.28	0.026			
**Mod. BNT**	FDG	Left inferior temporal gyrus	0.410	4.032	<0.001	9.494	0.408	<0.001
	PiB	Left caudal anterior cingulate cortex	0.546	3.619	0.001			
	PiB	Right precuneus	-1.196	-3.632	0.001			
	PiB	Left precuneus	0.895	2.641	0.010			
	FDG	Right insular lobe	-0.300	-2.802	0.007			
	FDG	Left inferior frontal gyrus, pars triangularis	0.237	2.001	0.049			
**MMSE**	FDG	Left middle temporal gyrus	0.223	1.805	0.076	14.364	0.555	<0.001
	CTh	Left precuneus	0.239	2.997	0.004			
	FDG	Left rostral middle frontal gyrus	0.478	3.443	0.001			
	FDG	Left postcentral gyrus	-0.598	-4.592	<0.001			
	FDG	Right caudal middle frontal gyrus	0.417	3.12	0.003			
	FDG	Right inferior frontal gyrus, pars orbitalis	-0.267	-2.65	0.010			
	FDG	Left fusiform gyrus	0.251	2.117	0.038			
**Word List—Immediate Recall**	FDG	Left middle temporal gyrus	0.569	6.665	<0.001	15.276	0.491	<0.001
	PiB	Left precuneus	-0.589	-4.655	<0.001			
	CTh	Right parahippocampal gyrus	-0.374	-4.239	<0.001			
	CTh	Right transverse temporal gyrus	0.276	3.156	0.002			
	PiB	Right postcentral gyrus	0.360	2.839	0.006			
**Word List—Delayed Recall**	CTh	Left inferior parietal lobule	0.520	4.619	<0.001	16.233	0.622	<0.001
	CTh	Right supramarginal gyrus	-1.011	-6.922	<0.001			
	CTh	Right posterior cingulate gyrus	0.387	4.183	<0.001			
	PiB	Right caudal middle frontal gyrus	0.676	4.738	<0.001			
	CTh	Right inferior parietal lobule	0.408	2.577	0.012			
	PiB	Right inferior parietal lobule	-0.565	-3.896	<0.001			
	PiB	Left insular lobe	-0.372	-2.466	0.016			
	CTh	Right caudal middle frontal gyrus	0.247	2.126	0.037			
**Word List—Savings**	CTh	Left precuneus	0.591	5.291	<0.001	10.97	0.353	<0.001
	CTh	Left postcentral gyrus	-0.407	-3.665	<0.001			
	PiB	Right inferior frontal gyrus, pars orbitalis	-0.674	-3.306	0.002			
	PiB	Right inferior frontal gyrus, pars triangularis	0.529	2.608	0.011			
**World List—Discriminability**	FDG	Left isthmus of the cingulate gyrus	0.349	3.296	0.002	7.434	0.216	<0.001
	CTh	Left insular lobe	0.523	3.311	0.001			
	CTh	Right superior frontal gyrus	-0.319	-2.022	0.047			
**Constructional Praxis**	FDG	Right precuneus	0.410	4.436	<0.001	13.189	0.565	<0.001
	CTh	Left fusiform gyrus	0.273	2.279	0.026			
	CTh	Right superior frontal gyrus	-0.879	-5.802	<0.001			
	FDG	Right medial orbitofrontal gyrus	-0.290	-3.231	0.002			
	CTh	Left posterior cingulate cortex	0.340	3.429	0.001			
	CTh	Right superior temporal gyrus	0.390	2.856	0.006			
	CTh	Right inferior frontal gyrus, pars orbitalis	0.319	2.315	0.024			
	CTh	Left entorhinal area	-0.201	-2.159	0.034			
**Figures—Recall**	FDG	Right precuneus	0.402	3.942	<0.001	12.535	0.235	<0.001
	CTh	Left cuneus	0.253	2.482	0.015			
**Figures—Savings**	FDG	Right isthmus of the cingulate gyrus	0.368	3.579	0.001	8.296	0.228	<0.001
	CTh	Left cuneus	0.266	2.563	0.013			
	PiB	Left transverse temporal gyrus	0.225	2.176	0.033			

Most predictive set of ROI-based biomarkers across modalities and cognitive performance as measured by z-scores on CERAD-NAB subtests.

β, standardized regression coefficient; CERAD-NAB, Consortium to Establish a Registry for Alzheimer’s Disease—Neuropsychological Assessment Battery; Corr., corrected; CTh, Cortical Thickness; FDG, [18F]-fluorodeoxyglucose; MMSE, Mini-Mental State Examination; PiB, Pittsburgh Compound B; ROI, region of interest.

#### Influence of Age and Disease Severity on Multiple Regression Analyses

In order to account for the possible influences of age and disease severity, age and CDR-SOB were forced as covariates into the regression models from *Prediction of Neuropsychological Performance by Cortical Amyloid Deposition* to *Prediction of Neuropsychological Performance by Regional Cortical Thickness*. Detailed results are given in the supplement (**Tables S6**-**S8**). The majority of beta coefficients remained rather stable. For a graphical overview of ROI-based correlations between age, disease-severity and the three imaging biomarkers, please see **Figure S1**.

In the models based on amyloid deposition, age was a significant predictor of performance at Word List—Savings (p=0.006). CDR-SOB was a significant predictor of Modified Boston Naming Test performance (p=0.024).

In the models based on glucose metabolism, age was a significant predictor of performance at Figures—Recall (p=0.042). CDR-SOB was a significant predictor of MMSE performance (p=0.006).

In the models based on cortical thickness, age was not a significant predictor of any cognitive subtest. CDR-SOB was a significant predictor of MMSE performance (p<0.001) and Figures—Savings (p=0.033).

## Discussion

In this study, we have systematically investigated the relationship between cognitive performance and three cortical imaging biomarkers, namely cortical thickness, glucose metabolism, and amyloid deposition in a single, reasonably sized cohort of well-characterized early AD patients.

We found that on a global level, only glucose metabolism but not cortical atrophy or cortical amyloid deposition was correlated with CERAD-NAB subtest results. Furthermore, regional glucose metabolism was able to explain the highest percentage of variance of neuropsychological test scores, followed by neurodegeneration measured by cortical thickness. Regression analyses of regional amyloid deposition predicting CERAD-NAB subtest performance were significant in 50% of subtests and explained the least percentages of test score variance.

Interestingly, regarding the most significant associations between cerebral ROIs and CERAD-NAB subtest scores, there is very little spatial agreement between cortical thickness and local glucose metabolism. With regard to cortical thickness, the majority of single ROIs with the highest regression coefficients is located in the medial and lateral parietal lobe. In contrast, the highest regression coefficients between glucose metabolism and CERAD-NAB subtest scores can be found both in the lateral temporal lobe and the medial parietal lobe. Also, a lateralization of glucose metabolism is associated with visuoconstructive subtests to the right parietal lobe, whereas subtests that predominantly check verbal domains are associated with glucose metabolism mostly in left temporal ROIs. This is in line with previous studies on the cerebral representation of CERAD subtests (30, 31). In our study, FDG uptake was rather closely associated to the physiological representations of cognitive domains while neuronal injury follows more the general distribution of AD in the inferior temporal lobe and the medial and lateral parietal lobe (3). In any case, there is a clear discrepancy in the spatial patterns of glucose metabolism and cortical thickness predicting cognitive functioning. In the currently proposed research framework both FDG-PET and structural MRI are considered biomarkers of neurodegeneration based on an assumed sequence of hypometabolism and neuron cell loss (5). However, our study suggests that these two modalities do not reflect the same aspects of neurodegeneration but on the contrary differ quite a lot spatially when predicting cognitive function in AD patients.

We reported significant predictions of cognitive function in half of CERAD-NAB subtests by cortical amyloid deposition in our cohort of early, but symptomatic AD patients. Furthermore, variance in regional amyloid deposition was higher than those of regional glucose metabolism and cortical thickness. This is remarkable because it is challenging concepts that propose a saturated state of amyloid deposition once AD patients become symptomatic (12). In contrary, our study suggests local amyloid burden measured by PiB-PET may at least in part be related to cognitive decline in patients with symptomatic early AD. This association has been shown in healthy older adults before (32, 33) and should encourage further investigation of regional quantification of cortical amyloid burden in the work-up of AD patients.

Interestingly, when forcing age and CDR-SOB (as a measure of disease severity) into the regression model, we found that these factors were significant only for very few cognitive subtests and that beta coefficients of biomarkers remained largely unchanged. The significant association between CDR-SOB and MMSE performance in the FDG-PET and cortical thickness based models stands out in this regard, which can be explained by the obvious association between increasing disease severity and poorer scores at the MMSE. Overall, we conclude that the influence of age and CDR-SOB as confounders to our analysis is rather small.

When including multimodal regional biomarker expression into the regression model, we found that the explained variance increased compared to unimodal regression models and that the remaining variables came from different regions and different biomarkers. This underscores the complex spatial relationship between brain regions and their biomarker expression. Future studies should focus on how regional biomarkers influence each other, e.g. by means of mediation analyses. The same is true for the multiple ROI approach compared to the single ROI approach, underlining the network character of AD pathophysiology.

Strengths of our study include the relatively large and well-characterized patient cohort, which was investigated by structural MRI, FDG-PET, and PiB-PET. Thus, we could study the association between neuropsychological impairments and different aspects of AD, amyloid plaque deposition, neuronal metabolism and neurodegeneration.

Limitations of our study include the cross-sectional character and lack of healthy individuals as controls. On the one hand, the selected ROI-based approach might be considered a limitation since it decreases the resolution and otherwise highly significant focal effects might be canceled out in large ROIs. On the other hand, we obtained identical spatial resolutions for the statistical comparisons for all imaging modalities by choosing a ROI approach. However, the impact of partial volume effects on ROI means due to different original resolutions of the imaging modalities cannot be ruled out and constitute a methodological limitation of the current study. Specifically, partial volume effects may be in part the reason for relatively diverging results of glucose metabolism and cortical thickness.

In conclusion, our study shows a tight association between FDG metabolism and physiological representations of neuropsychological capacities, while neurodegeneration could be observed mostly in areas that are generally affected during the course of AD. Moreover, we have shown that cortical amyloid deposition is predictive of cognitive functioning in half of CERAD-NAB subtests. This suggests direct or indirect functional relevance of cortical amyloid deposition in already symptomatic AD patients, which should encourage further investigation of regional amyloid quantification in symptomatic AD patients. Our results emphasize the complex spatial relationships between imaging biomarkers in AD and their different impact on cognitive functioning of early AD patients. Further studies are needed to elucidate the interaction of different biomarkers and their effect on cognitive functioning in early AD patients.

## Data Availability Statement

The datasets for this manuscript are not publicly available because: Restrictions regarding data availability were imposed by the local ethics committee. Requests to access the datasets should be directed to TG (t.grimmer@tum.de).

## Ethics Statement

The studies involving human participants were reviewed and approved by Ethics Committee of the School of Medicine of the Technical University of Munich. The patients/participants provided their written informed consent to participate in this study.

## Author Contributions

RD, TJ, IY, JD-S, and TG designed the experiment. RD, OG, MO, FM-S, and JD-S carried it out. DH, RD, OG, TG, and IY analyzed the data. DH, RD, and TG wrote the manuscript. TJ, IY, HF, JD-S, and CZ edited the manuscript. HF, JD-S, CZ, and TJ supervised the work. All authors discussed the results and reviewed the manuscript.

## Conflict of Interest

IY has no related conflicts of interest, outside the submitted work. IY is a consultant for Blue Earth Diagnostics and a lecturer for Piramal and has received grants from the Alzheimer Research Initiative Germany and the German Research Foundation (DFG). TG has no related conflicts of interest. Outside the submitted work, he reported having received consulting fees from Actelion, Biogen, Eli Lilly, Iqvia/Quintiles; MSD; Novartis, Quintiles, Roche Pharma, lecture fees from Biogen, Lilly, Parexel, Roche Pharma, and grants to his institution from Actelion, Novartis and PreDemTech.

The remaining authors declare that the research was conducted in the absence of any commercial or financial relationships that could be construed as a potential conflict of interest.
