# Supplementary material for: Regional Cerebral Associations Between Psychometric Tests and Imaging Biomarkers in Alzheimer’s Disease
Source: Front Psychiatry. 2020 Aug 13;11:793. doi: 10.3389/fpsyt.2020.00793 (PMC7438836; doi:10.3389/fpsyt.2020.00793)
Supplement: Supplementary file 1 [file DataSheet_1.docx]

Table S1: Regional amyloid uptake on PIB-PET

| **Region** | **Mean** | **SD** | **CV** | **Min** | **Max** |
| --- | --- | --- | --- | --- | --- |
| Right caudal anterior cingulate | 1.896 | 0.372 | 0.196 | 0.962 | 2.545 |
| Right caudal middle frontal | 1.832 | 0.324 | 0.177 | 1.090 | 2.429 |
| Right cuneus | 1.573 | 0.277 | 0.176 | 0.899 | 2.279 |
| Right entorhinal | 1.221 | 0.186 | 0.152 | 0.874 | 1.734 |
| Right fusiform | 1.674 | 0.277 | 0.165 | 0.845 | 2.388 |
| Right inferior parietal | 1.837 | 0.349 | 0.190 | 0.668 | 2.426 |
| Right inferior temporal | 1.687 | 0.337 | 0.200 | 0.779 | 2.630 |
| Right isthmus cingulate | 1.963 | 0.342 | 0.174 | 0.911 | 2.633 |
| Right lateral occipital | 1.433 | 0.294 | 0.205 | 0.536 | 2.237 |
| Right lateral orbitofrontal | 1.641 | 0.356 | 0.217 | 0.901 | 2.515 |
| Rightl ingual | 1.479 | 0.232 | 0.157 | 1.033 | 2.261 |
| Right medial orbitofrontal | 1.779 | 0.404 | 0.227 | 0.780 | 2.622 |
| Right middle temporal | 1.794 | 0.366 | 0.204 | 0.803 | 2.743 |
| Right parahippocampal | 1.416 | 0.214 | 0.151 | 0.908 | 2.011 |
| Right paracentral | 1.790 | 0.298 | 0.167 | 0.978 | 2.432 |
| Right pars opercularis | 1.800 | 0.343 | 0.191 | 1.009 | 2.474 |
| Right pars orbitalis | 1.666 | 0.356 | 0.213 | 0.940 | 2.763 |
| Right pars triangularis | 1.838 | 0.350 | 0.191 | 0.995 | 2.493 |
| Right pericalcarine | 1.693 | 0.326 | 0.193 | 1.047 | 2.584 |
| Right postcentral | 1.580 | 0.276 | 0.175 | 0.964 | 2.206 |
| Right posterior cingulate | 1.987 | 0.360 | 0.181 | 1.060 | 2.866 |
| Right precentral | 1.595 | 0.246 | 0.155 | 1.026 | 2.084 |
| Right precuneus | 2.056 | 0.390 | 0.190 | 0.789 | 2.964 |
| Right rostral anterior cingulate | 1.990 | 0.417 | 0.210 | 0.948 | 2.638 |
| Right rostral middle frontal | 1.879 | 0.445 | 0.237 | 0.891 | 2.803 |
| Right superior frontal | 1.807 | 0.384 | 0.212 | 0.899 | 2.525 |
| Right superior parietal | 1.759 | 0.337 | 0.192 | 0.702 | 2.368 |
| Right superior temporal | 1.651 | 0.336 | 0.204 | 0.943 | 2.356 |
| Right supremarginal | 1.786 | 0.334 | 0.187 | 0.893 | 2.687 |
| Right transverse temporal | 1.625 | 0.362 | 0.223 | 0.706 | 2.322 |
| Right insula | 1.744 | 0.350 | 0.201 | 0.994 | 2.582 |
| Left caudal anterior cingulate | 1.935 | 0.407 | 0.211 | 0.879 | 2.663 |
| Left caudal middle frontal | 1.878 | 0.355 | 0.189 | 1.112 | 2.669 |
| Left cuneus | 1.505 | 0.274 | 0.182 | 0.801 | 2.144 |
| Left enthorinal | 1.170 | 0.189 | 0.162 | 0.757 | 1.581 |
| Left fusiform | 1.694 | 0.281 | 0.166 | 0.904 | 2.357 |
| Left inferior parietal | 1.837 | 0.367 | 0.200 | 0.813 | 2.466 |
| Left inferior temporal | 1.705 | 0.342 | 0.201 | 0.873 | 2.542 |
| Left isthmus cingulate | 1.935 | 0.351 | 0.181 | 1.051 | 2.718 |
| Left lateral occipital | 1.490 | 0.293 | 0.197 | 0.689 | 2.299 |
| Left lateral orbitofrontal | 1.663 | 0.363 | 0.218 | 0.932 | 2.496 |
| Left lingual | 1.490 | 0.240 | 0.161 | 1.074 | 2.119 |
| Leftmedial orbitofrontal | 1.675 | 0.400 | 0.238 | 0.814 | 2.583 |
| Left Right middle temporal | 1.863 | 0.379 | 0.203 | 0.915 | 2.654 |
| Left parhippocampal | 1.364 | 0.219 | 0.160 | 0.890 | 1.853 |
| Left paracentral | 1.714 | 0.320 | 0.187 | 0.827 | 2.593 |
| Left pars opercularis | 1.828 | 0.367 | 0.201 | 0.988 | 2.564 |
| Left pars orbitalis | 1.764 | 0.358 | 0.203 | 1.011 | 2.648 |
| Left parstriangularis | 1.874 | 0.383 | 0.204 | 1.012 | 2.629 |
| Left pericalcarine | 1.651 | 0.335 | 0.203 | 1.051 | 2.425 |
| Left postcentral | 1.617 | 0.292 | 0.181 | 0.985 | 2.187 |
| Left posterior cingulate | 1.972 | 0.360 | 0.183 | 1.092 | 2.749 |
| Left precentral | 1.649 | 0.263 | 0.160 | 1.160 | 2.263 |
| Left precuneus | 2.013 | 0.393 | 0.195 | 0.918 | 2.854 |
| Left rostral anterior cingulate | 2.003 | 0.453 | 0.226 | 0.922 | 2.763 |
| Left rostral middle frontal | 1.899 | 0.454 | 0.239 | 0.907 | 2.828 |
| Left superior frontal | 1.746 | 0.391 | 0.224 | 0.868 | 2.476 |
| Left superior parietal | 1.786 | 0.340 | 0.190 | 0.850 | 2.386 |
| Left superior temporal | 1.666 | 0.343 | 0.206 | 0.958 | 2.404 |
| Left supramarginal | 1.846 | 0.348 | 0.189 | 0.960 | 2.610 |
| Left transverse temporal | 1.667 | 0.371 | 0.223 | 0.960 | 2.648 |
| Left insula | 1.731 | 0.345 | 0.199 | 1.030 | 2.452 |

Table S1: Regional amyloid uptake on PIB-PET. Signal intensities of [^11^C] PiB imaging data were normalized to the vermis cerebelli and are presented as SUVR.

Abbreviations SD: standard deviation; CV: coefficient of variation; Min: minimum; Max: maximum; PiB: Pittsburgh Compound B; PET: Positron emission tomography; SUVR: standardized uptake value ratio.

Table S2: Regional glucose metabolism on FDG-PET

| **Region** | **Mean** | **SD** | **CV** | **Min** | **Max** |
| --- | --- | --- | --- | --- | --- |
| Right caudal anterior cingulate | 1.350 | 0.131 | 0.097 | 1.011 | 1.682 |
| Right caudal middle frontal | 1.520 | 0.227 | 0.150 | 1.061 | 2.174 |
| Right cuneus | 1.582 | 0.200 | 0.126 | 1.098 | 2.122 |
| Right entorhinal | 1.034 | 0.134 | 0.130 | 0.696 | 1.326 |
| Right fusiform | 1.367 | 0.176 | 0.128 | 0.928 | 1.752 |
| Right inferior parietal | 1.270 | 0.217 | 0.171 | 0.815 | 1.731 |
| Right inferior temporal | 1.207 | 0.167 | 0.138 | 0.797 | 1.533 |
| Right isthmus cingulate | 1.444 | 0.221 | 0.153 | 1.060 | 2.299 |
| Right lateral occipital | 1.247 | 0.216 | 0.174 | 0.654 | 1.717 |
| Right lateral orbitofrontal | 1.344 | 0.164 | 0.122 | 0.948 | 1.904 |
| Rightl ingual | 1.573 | 0.181 | 0.115 | 1.102 | 2.035 |
| Right medial orbitofrontal | 1.342 | 0.161 | 0.120 | 0.872 | 1.866 |
| Right middle temporal | 1.272 | 0.181 | 0.142 | 0.877 | 1.667 |
| Right parahippocampal | 1.168 | 0.115 | 0.098 | 0.943 | 1.525 |
| Right paracentral | 1.529 | 0.138 | 0.090 | 1.302 | 1.888 |
| Right pars opercularis | 1.522 | 0.163 | 0.107 | 1.138 | 2.009 |
| Right pars orbitalis | 1.386 | 0.209 | 0.150 | 0.767 | 1.876 |
| Right pars triangularis | 1.448 | 0.178 | 0.123 | 0.899 | 1.765 |
| Right pericalcarine | 1.723 | 0.211 | 0.123 | 1.265 | 2.272 |
| Right postcentral | 1.445 | 0.167 | 0.115 | 1.045 | 1.887 |
| Right posterior cingulate | 1.477 | 0.159 | 0.108 | 1.109 | 1.968 |
| Right precentral | 1.506 | 0.166 | 0.110 | 1.081 | 1.902 |
| Right precuneus | 1.510 | 0.229 | 0.152 | 0.963 | 2.267 |
| Right rostral anterior cingulate | 1.352 | 0.148 | 0.109 | 0.901 | 1.810 |
| Right rostral middle frontal | 1.441 | 0.257 | 0.178 | 0.704 | 1.865 |
| Right superior frontal | 1.422 | 0.191 | 0.135 | 0.888 | 1.944 |
| Right superior parietal | 1.318 | 0.210 | 0.159 | 0.836 | 1.755 |
| Right superior temporal | 1.239 | 0.137 | 0.110 | 0.956 | 1.593 |
| Right supremarginal | 1.339 | 0.185 | 0.138 | 0.883 | 1.797 |
| Right transverse temporal | 1.560 | 0.204 | 0.131 | 1.041 | 2.086 |
| Right insula | 1.402 | 0.117 | 0.084 | 1.123 | 1.720 |
| Left caudal anterior cingulate | 1.411 | 0.142 | 0.101 | 1.059 | 1.775 |
| Left caudal middle frontal | 1.494 | 0.225 | 0.151 | 0.980 | 2.024 |
| Left cuneus | 1.531 | 0.198 | 0.130 | 1.004 | 1.926 |
| Left enthorinal | 0.994 | 0.119 | 0.120 | 0.570 | 1.262 |
| Left fusiform | 1.345 | 0.167 | 0.124 | 0.923 | 1.716 |
| Left inferior parietal | 1.264 | 0.213 | 0.168 | 0.728 | 1.640 |
| Left inferior temporal | 1.174 | 0.160 | 0.136 | 0.755 | 1.526 |
| Left isthmus cingulate | 1.457 | 0.209 | 0.143 | 1.043 | 2.123 |
| Left lateral occipital | 1.296 | 0.222 | 0.171 | 0.639 | 1.741 |
| Left lateral orbitofrontal | 1.334 | 0.174 | 0.131 | 0.747 | 1.856 |
| Left lingual | 1.577 | 0.170 | 0.108 | 1.201 | 1.962 |
| Leftmedial orbitofrontal | 1.290 | 0.177 | 0.137 | 0.843 | 1.745 |
| Left Right middle temporal | 1.262 | 0.177 | 0.140 | 0.826 | 1.625 |
| Left parhippocampal | 1.131 | 0.101 | 0.089 | 0.914 | 1.369 |
| Left paracentral | 1.548 | 0.156 | 0.101 | 1.233 | 1.972 |
| Left pars opercularis | 1.490 | 0.157 | 0.105 | 1.153 | 1.934 |
| Left pars orbitalis | 1.406 | 0.195 | 0.139 | 0.787 | 1.747 |
| Left parstriangularis | 1.448 | 0.201 | 0.139 | 0.891 | 1.817 |
| Left pericalcarine | 1.723 | 0.229 | 0.133 | 1.229 | 2.201 |
| Left postcentral | 1.466 | 0.162 | 0.111 | 1.046 | 1.887 |
| Left posterior cingulate | 1.483 | 0.164 | 0.110 | 1.161 | 1.944 |
| Left precentral | 1.502 | 0.159 | 0.106 | 1.142 | 1.856 |
| Left precuneus | 1.503 | 0.231 | 0.153 | 0.928 | 2.170 |
| Left rostral anterior cingulate | 1.418 | 0.157 | 0.111 | 0.898 | 1.901 |
| Left rostral middle frontal | 1.421 | 0.245 | 0.172 | 0.541 | 1.845 |
| Left superior frontal | 1.371 | 0.203 | 0.148 | 0.692 | 1.814 |
| Left superior parietal | 1.310 | 0.180 | 0.138 | 0.796 | 1.620 |
| Left superior temporal | 1.228 | 0.135 | 0.110 | 0.891 | 1.495 |
| Left supramarginal | 1.351 | 0.175 | 0.130 | 0.944 | 1.699 |
| Left transverse temporal | 1.584 | 0.198 | 0.125 | 1.026 | 1.997 |
| Left insula | 1.363 | 0.111 | 0.082 | 1.028 | 1.644 |

Table S2: Regional glucose metabolism on FDG-PET. Signal intensities of [^18^F] FDG imaging data were normalized to the pons.

Abbreviations SD: standard deviation; CV: coefficient of variation; FDG: [18F]-fluorodeoxyglucose; Min: minimum; Max: maximum, PET: Positron emission tomography.

Table S3: Regional cortical thickness on structural MRI

| **Region** | **Mean** | **SD** | **CV** | **Min** | **Max** |
| --- | --- | --- | --- | --- | --- |
| Right caudal anterior cingulate | 2.49 | 0.35 | 0.140 | 1.67 | 3.53 |
| Right caudal middle frontal | 2.29 | 0.25 | 0.110 | 1.70 | 2.96 |
| Right cuneus | 1.72 | 0.18 | 0.106 | 1.28 | 2.33 |
| Right entorhinal | 3.03 | 0.63 | 0.209 | 0.00 | 4.35 |
| Right fusiform | 2.33 | 0.28 | 0.120 | 1.61 | 3.00 |
| Right inferior parietal | 2.14 | 0.23 | 0.108 | 1.58 | 2.89 |
| Right inferior temporal | 2.50 | 0.26 | 0.103 | 1.97 | 3.28 |
| Right isthmus cingulate | 2.12 | 0.32 | 0.150 | 1.40 | 3.14 |
| Right lateral occipital | 1.98 | 0.21 | 0.108 | 1.41 | 2.52 |
| Right lateral orbitofrontal | 2.45 | 0.24 | 0.096 | 1.77 | 3.13 |
| Rightl ingual | 1.82 | 0.18 | 0.100 | 1.36 | 2.37 |
| Right medial orbitofrontal | 2.31 | 0.25 | 0.107 | 1.59 | 2.92 |
| Right middle temporal | 2.52 | 0.27 | 0.107 | 1.91 | 3.29 |
| Right parahippocampal | 2.31 | 0.42 | 0.182 | 0.00 | 2.96 |
| Right paracentral | 2.13 | 0.23 | 0.109 | 1.43 | 2.58 |
| Right pars opercularis | 2.33 | 0.21 | 0.092 | 1.64 | 2.96 |
| Right pars orbitalis | 2.48 | 0.28 | 0.111 | 1.51 | 3.07 |
| Right pars triangularis | 2.26 | 0.23 | 0.101 | 1.61 | 2.78 |
| Right pericalcarine | 1.52 | 0.17 | 0.114 | 1.14 | 2.11 |
| Right postcentral | 1.84 | 0.21 | 0.112 | 1.38 | 2.47 |
| Right posterior cingulate | 2.29 | 0.24 | 0.107 | 1.43 | 2.78 |
| Right precentral | 2.23 | 0.26 | 0.116 | 1.51 | 2.72 |
| Right precuneus | 2.05 | 0.23 | 0.111 | 1.37 | 2.87 |
| Right rostral anterior cingulate | 2.78 | 0.32 | 0.116 | 1.61 | 3.61 |
| Right rostral middle frontal | 2.16 | 0.22 | 0.100 | 1.73 | 2.90 |
| Right superior frontal | 2.42 | 0.23 | 0.096 | 1.70 | 2.95 |
| Right superior parietal | 1.96 | 0.24 | 0.124 | 1.42 | 2.96 |
| Right superior temporal | 2.39 | 0.25 | 0.106 | 1.84 | 3.07 |
| Right supremarginal | 2.22 | 0.24 | 0.109 | 1.50 | 2.81 |
| Right transverse temporal | 2.07 | 0.31 | 0.149 | 1.16 | 2.78 |
| Right insula | 2.78 | 0.30 | 0.107 | 1.55 | 3.39 |
| Left caudal anterior cingulate | 2.57 | 0.32 | 0.123 | 1.76 | 3.31 |
| Left caudal middle frontal | 2.24 | 0.23 | 0.103 | 1.67 | 2.74 |
| Left cuneus | 1.71 | 0.18 | 0.104 | 1.35 | 2.29 |
| Left enthorinal | 2.85 | 0.48 | 0.169 | 1.29 | 3.93 |
| Left fusiform | 2.34 | 0.24 | 0.104 | 1.71 | 3.17 |
| Left inferior parietal | 2.09 | 0.22 | 0.107 | 1.50 | 2.70 |
| Left inferior temporal | 2.45 | 0.30 | 0.120 | 1.96 | 3.29 |
| Left isthmus cingulate | 2.12 | 0.29 | 0.136 | 1.46 | 3.05 |
| Left lateral occipital | 1.93 | 0.22 | 0.112 | 1.40 | 2.50 |
| Left lateral orbitofrontal | 2.43 | 0.24 | 0.101 | 1.88 | 3.40 |
| Left lingual | 1.77 | 0.16 | 0.091 | 1.39 | 2.45 |
| Left medial orbitofrontal | 2.26 | 0.24 | 0.106 | 1.62 | 2.92 |
| Left middle temporal | 2.43 | 0.29 | 0.118 | 1.52 | 3.23 |
| Left parhippocampal | 2.37 | 0.29 | 0.121 | 1.75 | 2.90 |
| Left paracentral | 2.15 | 0.26 | 0.120 | 1.34 | 2.90 |
| Left pars opercularis | 2.28 | 0.23 | 0.102 | 1.61 | 2.77 |
| Left pars orbitalis | 2.39 | 0.27 | 0.111 | 1.59 | 3.04 |
| Left parstriangularis | 2.20 | 0.19 | 0.089 | 1.83 | 2.70 |
| Left pericalcarine | 1.49 | 0.15 | 0.102 | 1.15 | 2.03 |
| Left postcentral | 1.84 | 0.19 | 0.101 | 1.41 | 2.35 |
| Left posterior cingulate | 2.28 | 0.26 | 0.112 | 1.52 | 2.88 |
| Left precentral | 2.22 | 0.24 | 0.108 | 1.49 | 2.71 |
| Left precuneus | 2.02 | 0.22 | 0.109 | 1.44 | 2.69 |
| Left rostral anterior cingulate | 2.73 | 0.35 | 0.129 | 1.40 | 3.51 |
| Left rostral middle frontal | 2.11 | 0.20 | 0.096 | 1.57 | 2.63 |
| Left superior frontal | 2.41 | 0.23 | 0.094 | 1.78 | 2.86 |
| Left superior parietal | 1.94 | 0.22 | 0.114 | 1.50 | 2.69 |
| Left superior temporal | 2.33 | 0.24 | 0.103 | 1.69 | 2.86 |
| Left supramarginal | 2.17 | 0.21 | 0.097 | 1.69 | 2.72 |
| Left transverse temporal | 2.03 | 0.31 | 0.152 | 1.18 | 2.65 |
| Left insula | 2.80 | 0.28 | 0.099 | 1.92 | 3.29 |

Table S3: Regional cortical thickness on structural MRI.

Abbreviations SD: standard deviation; CV: coefficient of variation; FDG: [18F]-fluorodeoxyglucose; Min: minimum; Max: maximum, MRI: Magnetic resonance imaging.

Table S4: Correlation of neuropsychological test scores with regional glucose metabolism.

| **CERAD-NAB subtest** | **ROI** | **r** | **p*** |
| --- | --- | --- | --- |
| **Verbal Fluency** | Left middle temporal gyrus | 0.506 | <0.001 |
|  | Left inferor temporal gyrus | 0.476 | 0.001 |
|  | Left inferior parietal lobule | 0.438 | 0.005 |
|  | Left superior temporal gyrus | 0.429 | 0.008 |
| **Modified Boston Naming Test** | Left inferior temporal gyrus | 0.433 | 0.006 |
| **MMSE** | Right caudal middle frontal gyrus | 0.425 | 0.008 |
|  | Right rostral middle frontal gyrus | 0.417 | 0.011 |
|  | Right inferior parietal lobule | 0.378 | 0.048 |
|  | Left middle temporal gyrus | 0.556 | 0.000 |
|  | Left caudal middle frontal gyrus | 0.499 | 0.000 |
|  | Left rostral middle frontal gyrus | 0.497 | 0.000 |
|  | Left superior temporal gyrus | 0.475 | 0.001 |
|  | Left inferior parietal lobule | 0.458 | 0.002 |
|  | Left inferior temporal gyrus | 0.449 | 0.003 |
|  | Left inferior frontal gyrus pars triangularis | 0.443 | 0.004 |
|  | Left supramarginal gyrus | 0.440 | 0.004 |
|  | Left inferior frontal gyrus. pars opercularis | 0.409 | 0.015 |
|  | Left transverse temporal gyrus | 0.402 | 0.019 |
|  | Left superior frontal gyrus | 0.395 | 0.025 |
|  | Left isthmus of the cingulate cortex | 0.395 | 0.026 |
|  | Left precuneus | 0.391 | 0.029 |
|  | Left fusiform gyrus | 0.389 | 0.032 |
| **Word List – Immediate Recall** | Left middle temporal gyrus | 0.473 | 0.001 |
|  | Left inferior frontal gyrus pars triangularis | 0.464 | 0.002 |
|  | Left rostral middle frontal gyrus | 0.457 | 0.002 |
|  | Left caudal middle frontal gyrus | 0.456 | 0.002 |
|  | Left superior temporal gyrus | 0.404 | 0.020 |
|  | Left inferior parietal lobule | 0.394 | 0.029 |
|  | Left inferior frontal gyrus pars opercularis | 0.393 | 0.030 |
| **Word List - Delayed Recall** | None |  |  |
| **Word List - Savings** | None |  |  |
| **World List - Discriminability** | None |  |  |
| **Constructional Praxis** | Right precuneus | 0.464 | 0.001 |
|  | Right posterior cingulate cortex | 0.420 | 0.010 |
|  | Right transverse temporal gyrus | 0.380 | 0.044 |
|  | Left posterior cingulate cortex | 0.379 | 0.046 |
|  | Left precuneus | 0.378 | 0.047 |
| **Figures - Recall** | Right precuneus | 0.439 | 0.004 |
|  | Right isthmus of the cingulate cortex | 0.436 | 0.005 |
|  | Right cuneus cortex | 0.413 | 0.013 |
|  | Right posterior cingulate cortex | 0.402 | 0.020 |
|  | Right inferior parietal lobule | 0.393 | 0.028 |
|  | Right frontal middle gyrus | 0.382 | 0.041 |
|  | Left precuneus | 0.435 | 0.005 |
|  | Left inferior parietal lobule | 0.400 | 0.021 |
|  | Left isthmus of the cingulate gyrus | 0.392 | 0.028 |
|  | Left fusiform gyrus | 0.389 | 0.032 |
| **Figures - Savings** | Right isthmus of the cingulate gyrus | 0.393 | 0.001 |

Table S4: Correlation of neuropsychological test scores with regional glucose metabolism as measured by FDG-PET. Correlation coefficients, which remain significant after correction for multiple comparisons using the Bonferroni method, are stated. P-values are Bonferroni corrected (*) for testing 62 different brain regions. Abbreviations: CERAD-NAB: Consortium to Establish a Registry for Alzheimer’s Disease – Neuropsychological Assessment Battery, FDG: [18F]-fluorodeoxyglucose, MMSE: Mini-Mental State Examination, PET: Positron emission tomography.

Table S5: Correlation of neuropsychological test scores with regional cortical thickness.

| **CERAD-NAB subtest** | **ROI** | **r** | **p*** |
| --- | --- | --- | --- |
| **Verbal Fluency** | Left inferior parietal lobule | 0.381 | 0.046 |
| **Modified Boston Naming Test** | None |  |  |
| **MMSE** | Left precuneus | 0.395 | 0.025 |
| **Word List – Immediate Recall** | Left inferior parietal lobule | 0.411 | 0.016 |
| **Word List - Delayed Recall** | Left inferior parietal lobule | 0.438 | 0.005 |
|  | Left precuneus | 0.421 | 0.011 |
|  | Left posterior cingulate cortex | 0.397 | 0.026 |
| **Word List - Savings** | Left precuneus | 0.412 | 0.016 |
| **World List - Discriminability** | None |  |  |
| **Constructional Praxis** | Right fusiform gyrus | 0.407 | 0.016 |
|  | Right lingual gyrus | 0.401 | 0.021 |
|  | Left fusiform gyrus | 0.390 | 0.031 |
| **Figures - Recall** | None |  |  |
| **Figures - Savings** | None |  |  |

Table S5: Correlation of neuropsychological test scores with regional cortical thickness measured by structural MRI. Correlation coefficients, which remain significant after correction for multiple comparisons using the Bonferroni method, are stated. P-values are Bonferroni corrected (*) for testing 62 different brain regions.

Abbreviations: CERAD-NAB: Consortium to Establish a Registry for Alzheimer’s Disease – Neuropsychological Assessment Battery, MMSE: Mini-Mental State Examination, MRI: Magnetic resonance imaging.

Table S6: Relationship between cognitive performance and ROI-based amyloid deposition controlled for age and disease severity.

| **CERAD-NAB subtest** |  | **Set of ROIs** | **β** | **T** | **p*** | **F** | **Corr. R^2^** | **p** |
| --- | --- | --- | --- | --- | --- | --- | --- | --- |
| **Verbal Fluency** |  | *No significant ROI* |  |  |  |  |  |  |
| **Mod. BNT** |  | Left entorhinal cortex | 0.341 | 3.225 | 0.009 | 6.132 | 0.172 | 0.001 |
|  |  | Age | 0.341 | 0.288 | 1.000 |  |  |  |
|  |  | CDR-SOB | -0.291 | -2.739 | 0.024 |  |  |  |
| **MMSE** |  | *No significant ROI* |  |  |  |  |  |  |
| **Word List – Immediate Recall** |  | Left precuneus | -0.669 | -3.755 | <0.001 | 5.952 | 0.211 | <0.001 |
|  |  | Right fusiform gyrus | 0.458 | 2.527 | 0.042 |  |  |  |
|  |  | Age | 0.132 | 1.239 | 0.660 |  |  |  |
|  |  | CDR-SOB | -0.244 | -2.326 | 0.069 |  |  |  |
| **Word List - Delayed Recall** |  | Left precuneus | -0.718 | -3.701 | <0.001 | 7.896 | 0.220 | <0.001 |
|  |  | Left caudal middle frontal gyrus | 0.551 | 2.808 | 0.054 |  |  |  |
|  |  | Age | 1.604 | 0.113 | 0.339 |  |  |  |
|  |  | CDR-SOB | -0.192 | -1.851 | 0.204 |  |  |  |
| **Word List - Savings** |  | Right inferior frontal gyrus. pars orbitalis | -0.176 | -1.598 | 0.345 | 5.942 | 0.169 | 0.001 |
|  |  | Age | 0.341 | 3.166 | 0.006 |  |  |  |
|  |  | CDR-SOB | -0.145 | -1.313 | 0.579 |  |  |  |
| **World List - Discriminability** |  | *No significant ROI* |  |  |  |  |  |  |
| **Constructional Praxis** |  | *No significant ROI* |  |  |  |  |  |  |
| **Figures - Recall** |  | *No significant ROI* |  |  |  |  |  |  |
| **Figures - Savings** |  | Right caudal middle frontal gyrus | 0.688 | 3.467 | 0.003 | 8.060 | 0.220 | <0.001 |
|  |  | Right lateral orbitofrontal gyrus | -0.544 | -2.715 | 0.024 |  |  |  |
|  |  | Left parahippocampal gyrus | 0.206 | 1.796 | 0.231 |  |  |  |
|  |  | Age | 0.138 | 1.276 | 0.618 |  |  |  |
|  |  | CDR-SOB | -0.157 | -1.362 | 0.531 |  |  |  |

Table S6: Relationship between regional amyloid deposition as measured by [^11^C] PiB-PET and cognitive performance as measured by z-scores on CERAD-NAB subtests. Coefficients of determination are given for the set of most predictive ROIs with regard to CERAD-NAB subtests performance. Age and CDR-SOB are forced into the regression model. P-values are Bonferroni corrected (*) for testing three different biomarkers.

Abbreviations: β: standardized regression coefficient, CDR-SOB: Clinical Dementia Rating – Sum of boxes, CERAD-NAB: Consortium to Establish a Registry for Alzheimer’s Disease – Neuropsychological Assessment Battery, Corr.: corrected, MMSE: Mini-Mental State Examination, Mod. BNT: Modified Boston Naming Test, PET: Positron emission tomography, PIB: Pittsburgh Compound B, ROI: region of interest.

Table S7: Relationship between cognitive performance and ROI-based glucose metabolism controlled for age and disease severity.

| **CERAD-NAB subtest** | **Set of ROIs** | **β** | **T** | **p*** | **F** | **Corr. R^2^** | **p** |
| --- | --- | --- | --- | --- | --- | --- | --- |
| **Verbal Fluency** | Right parahippocampal gyrus | -0.481 | -5.044 | <0.001 | 12.606 | 0.440 | <0.001 |
|  | Left transverse temporal gyrus | 0.255 | 2.298 | 0.075 |  |  |  |
|  | Left inferior temporal gyrus | 0.413 | 3.965 | <0.001 |  |  |  |
|  | Age | 0.063 | 0.684 | 1.000 |  |  |  |
|  | CDR-SOB | -0.192 | -1.889 | 0.189 |  |  |  |
| **Mod. BNT** | Left inferior temporal gyrus | 0.415 | 3.848 | <0.001 | 6.703 | 0.278 | <0.001 |
|  | Right postcentral gyrus | -0.548 | -3.181 | 0.006 |  |  |  |
|  | Right superior frontal gyrus | 0.297 | 1.647 | 0.312 |  |  |  |
|  | Age | -0.037 | -0.365 | 1.000 |  |  |  |
|  | CDR-SOB | -0.209 | -1.815 | 0.222 |  |  |  |
| **MMSE** | Left middle temporal gyrus | 0.154 | 1.226 | 0.672 | 13.575 | 0.573 | <0.001 |
|  | Left rostral middle frontal gyrus | 0.518 | 3.395 | 0.003 |  |  |  |
|  | Left postcentral gyrus | -0.527 | -3.775 | <0.001 |  |  |  |
|  | Right caudal middle frontal gyrus | 0.375 | 2.085 | 0.123 |  |  |  |
|  | Right inferior frontal gyrus pars triangularis | -0.372 | -2.336 | 0.066 |  |  |  |
|  | Left fusiform gyrus | 0.248 | 2.054 | 0.132 |  |  |  |
|  | Age | 0.083 | 0.931 | 1.000 |  |  |  |
|  | CDR-SOB | -0.316 | -3.266 | 0.006 |  |  |  |
| **Word List – Immediate Recall** | Left middle temporal gyrus | 0.258 | 2.048 | 0.132 | 7.522 | 0.353 | <0.001 |
|  | Left rostral middle frontal gyrus | 0.639 | 3.493 | 0.003 |  |  |  |
|  | Right superior frontal gyrus | -0.464 | -2.755 | 0.024 |  |  |  |
|  | Age | 0.063 | 0.636 | 1.000 |  |  |  |
|  | CDR-SOB | -0.068 | -0.598 | 1.000 |  |  |  |
| **Word List - Delayed Recall** | Left isthmus of cingulate gyrus | 0.432 | 3.54 | 0.003 | 6.412 | 0.226 | <0.001 |
|  | Right insula | -0.345 | -2.933 | 0.015 |  |  |  |
|  | Age | 0.065 | 0.595 | 1.000 |  |  |  |
|  | CDR-SOB | -0.135 | -1.151 | 0.762 |  |  |  |
| **Word List - Savings** | Left isthmus of cingulate gyrus | 0.526 | 4.311 | <0.001 | 7.626 | 0.421 | <0.001 |
|  | Right medial orbitofrontal gyrus | 0.783 | 4.461 | <0.001 |  |  |  |
|  | Right lateral orbitofrontal gyrus | -0.783 | -4.180 | <0.001 |  |  |  |
|  | Left insula | -0.323 | -2.254 | 0.084 |  |  |  |
|  | Right lingual gyrus | -0.370 | -2.940 | 0.015 |  |  |  |
|  | Right precentral gyrus | 0.245 | 1.835 | 0.213 |  |  |  |
|  | Age | 0.193 | 1.926 | 0.174 |  |  |  |
|  | CDR-SOB | -0.080 | -0.772 | 1.000 |  |  |  |
| **World List - Discriminability** | Left isthmus of the cingulate gyrus | 0.394 | 3.074 | 0.009 | 4.630 | 0.206 | 0.001 |
|  | Right transverse temporal gyrus | -0.344 | -2.777 | 0.021 |  |  |  |
|  | Left entorhinal cortex | 0.214 | 1.948 | 0.168 |  |  |  |
|  | Age | -0.072 | -0.638 | 1.000 |  |  |  |
|  | CDR-SOB | -0.141 | -1.132 | 0.786 |  |  |  |
| **Constructional Praxis** | Right precuneus | 0.566 | 4.764 | <0.001 | 7.301 | 0.296 | <0.001 |
|  | Right medial orbitofrontal gyrus | -0.405 | -3.459 | 0.003 |  |  |  |
|  | Left inferior temporal gyrus | 0.258 | 2.297 | 0.075 |  |  |  |
|  | Age | -0.026 | -0.263 | 1.000 |  |  |  |
|  | CDR-SOB | -0.018 | -0.164 | 1.000 |  |  |  |
| **Figures - Recall** | Right precuneus | 0.304 | 2.679 | 0.027 | 9.490 | 0.254 | <0.001 |
|  | Age | 0.257 | 2.526 | 0.042 |  |  |  |
|  | CDR-SOB | -0.181 | -1.622 | 0.327 |  |  |  |
| **Figures - Savings** | Right isthmus of the cingulate gyrus | 0.220 | 1.782 | 0.237 | 5.439 | 0.194 | 0.001 |
|  | Left lateral occipital complex | 0.218 | 1.840 | 0.210 |  |  |  |
|  | Age | 0.125 | 1.173 | 0.735 |  |  |  |
|  | CDR-SOB | -0.158 | -1.345 | 0.549 |  |  |  |

Table S7: Relationship between regional glucose metabolism as measured by [^18^F] FDG-PET and cognitive performance as measured by z-scores on CERAD-NAB subtests. Coefficients of determination are given for the set of most predictive ROIs with regard to CERAD-NAB subtests performance. Age and CDR-SOB are forced into the regression model. P-values are Bonferroni corrected (*) for testing three different biomarkers.

Abbreviations: β: standardized regression coefficient. CDR-SOB: Clinical Dementia Rating – Sum of boxes. CERAD-NAB: Consortium to Establish a Registry for Alzheimer’s Disease – Neuropsychological Assessment Battery. Corr.: corrected. FDG: [18F]-fluorodeoxyglucose. MMSE: Mini-Mental State Examination. PET: Positron emission tomography. ROI: region of interest.

Table S8: Relationship between cognitive performance and ROI-based cortical thickness controlled for age and disease severity.

| **CERAD-NAB subtest** | **Set of ROIs** | **β** | **T** | **p*** | **F** | **Corr. R^2^** | **p** |
| --- | --- | --- | --- | --- | --- | --- | --- |
| **Verbal Fluency** | Left inferior parietal lobule | 0.322 | 1.86 | 0.201 | 8.779 | 0.424 | <0.001 |
|  | Left paracentral gyrus | -0.396 | -3.497 | 0.003 |  |  |  |
|  | Right lingual gyrus | -0.431 | -3.632 | 0.003 |  |  |  |
|  | Left superior temporal gyrus | 0.337 | 2.55 | 0.039 |  |  |  |
|  | Left precuneus | 0.266 | 1.522 | 0.399 |  |  |  |
|  | Age | 0.088 | 0.822 | 1.000 |  |  |  |
|  | CDR-SOB | -0.178 | -1.917 | 0.177 |  |  |  |
| **Mod. BNT** | Left fusiform gyrus | 0.433 | 3.257 | 0.006 | 4.644 | 0.165 | 0.002 |
|  | Right inferior frontal gyrus. pars orbitalis | -0.268 | -2.037 | 0.135 |  |  |  |
|  | Age | 0.019 | 0.178 | 1.000 |  |  |  |
|  | CDR-SOB | -0.162 | -1.404 | 0.495 |  |  |  |
| **MMSE** | Left precuneus | 0.428 | 3.582 | 0.003 | 13.030 | 0.490 | <0.001 |
|  | Right cuneus | -0.253 | -2.357 | 0.063 |  |  |  |
|  | Right entorhinal cortex | 0.055 | 0.559 | 1.000 |  |  |  |
|  | Left rostral anterior cingulate cortex | -0.147 | -1.648 | 0.312 |  |  |  |
|  | Age | 0.123 | 1.324 | 0.570 |  |  |  |
|  | CDR-SOB | -0.465 | -4.728 | <0.001 |  |  |  |
| **Word List – Immediate Recall** | Left parietal inferior lobule | 0.580 | 4.326 | <0.001 | 7.825 | 0.408 | <0.001 |
|  | Right supramarginal gyrus | -0.428 | -3.355 | 0.003 |  |  |  |
|  | Left medial orbitofrontal gyrus | -0.263 | -2.291 | 0.075 |  |  |  |
|  | Left posterior cingulate cortex | 0.294 | 2.358 | 0.063 |  |  |  |
|  | Age | -0.039 | -0.376 | 1.000 |  |  |  |
|  | CDR-SOB | -0.175 | -1.816 | 0.222 |  |  |  |
| **Word List - Delayed Recall** | Left parietal inferior lobule | 0.457 | 3.475 | 0.003 | 9.598 | 0.449 | <0.001 |
|  | Right supramarginal gyrus | -0.762 | -4.556 | <0.001 |  |  |  |
|  | Right parietal inferior lobule | 0.635 | 2.806 | 0.021 |  |  |  |
|  | Right lateral occipital complex | -0.437 | -3.261 | 0.006 |  |  |  |
|  | Right cuneus | 0.373 | 3.008 | 0.012 |  |  |  |
|  | Age | -0.021 | -0.213 | 1.000 |  |  |  |
|  | CDR-SOB | -0.129 | -1.345 | 0.552 |  |  |  |
| **Word List - Savings** | Left precuneus | 0.519 | 3.909 | <0.001 | 8.698 | 0.335 | <0.001 |
|  | Right postcentral gyrus | -0.325 | -2.544 | 0.039 |  |  |  |
|  | Age | 0.156 | 1.368 | 0.528 |  |  |  |
|  | CDR-SOB | -0.178 | -1.801 | 0.228 |  |  |  |
| **World List - Discriminability** | Left posterior cingulate cortex | 0.271 | 2.292 | 0.075 | 3.283 | 0.089 | 0.026 |
|  | Age | -0.014 | -0.122 | 1.000 |  |  |  |
|  | CDR-SOB | -0.197 | -1.699 | 0.282 |  |  |  |
| **Constructional Praxis** | Right fusiform gyrus | 0.239 | 1.828 | 0.216 | 10.835 | 0.512 | <0.001 |
|  | Right medial orbitofrontal gyrus | -0.343 | -3.019 | 0.012 |  |  |  |
|  | Right posterior cingulate cortex | 0.303 | 2.618 | 0.033 |  |  |  |
|  | Right superior frontal gyrus | -0.559 | -4.106 | <0.001 |  |  |  |
|  | Left isthmus of the cingulate gyrus | 0.367 | 3.628 | 0.003 |  |  |  |
|  | Right lingual gyrus | 0.443 | 3.448 | 0.003 |  |  |  |
|  | Age | -0.037 | -0.439 | 1.000 |  |  |  |
|  | CDR-SOB | -0.077 | -0.888 | 1.000 |  |  |  |
| **Figures - Recall** | Right inferior parietal lobule | 0.538 | 2.543 | 0.039 | 6.985 | 0.242 | <0.001 |
|  | Right supramarginal gyrus | -0.356 | -1.799 | 0.228 |  |  |  |
|  | Age | 0.182 | 1.633 | 0.321 |  |  |  |
|  | CDR-SOB | -0.242 | -2.292 | 0.075 |  |  |  |
| **Figures - Savings** | Left cuneus | 0.221 | 2.019 | 0.141 | 4.977 | 0.139 | 0.003 |
|  | Age | 0.1 | 0.92 | 1.000 |  |  |  |
|  | CDR-SOB | -0.289 | -2.628 | 0.033 |  |  |  |

Table S8: Relationship between regional cortical thickness as measured by structural MRI and cognitive performance as measured by z-scores on CERAD-NAB subtests. Coefficients of determination are given for the set of most predictive ROIs with regard to CERAD-NAB subtests performance. Age and CDR-SOB are forced into the regression model. P-values are Bonferroni corrected (*) for testing three different biomarkers.

Abbreviations: β: standardized regression coefficient, CDR-SOB: Clinical Dementia Rating – Sum of boxes, CERAD-NAB: Consortium to Establish a Registry for Alzheimer’s Disease – Neuropsychological Assessment Battery, Corr.: corrected, MMSE: Mini-Mental State Examination, MRI: Magnetic Resonance Imaging, ROI: region of interest.

Figure S1: Correlations between imaging biomarkers and age and disease severity


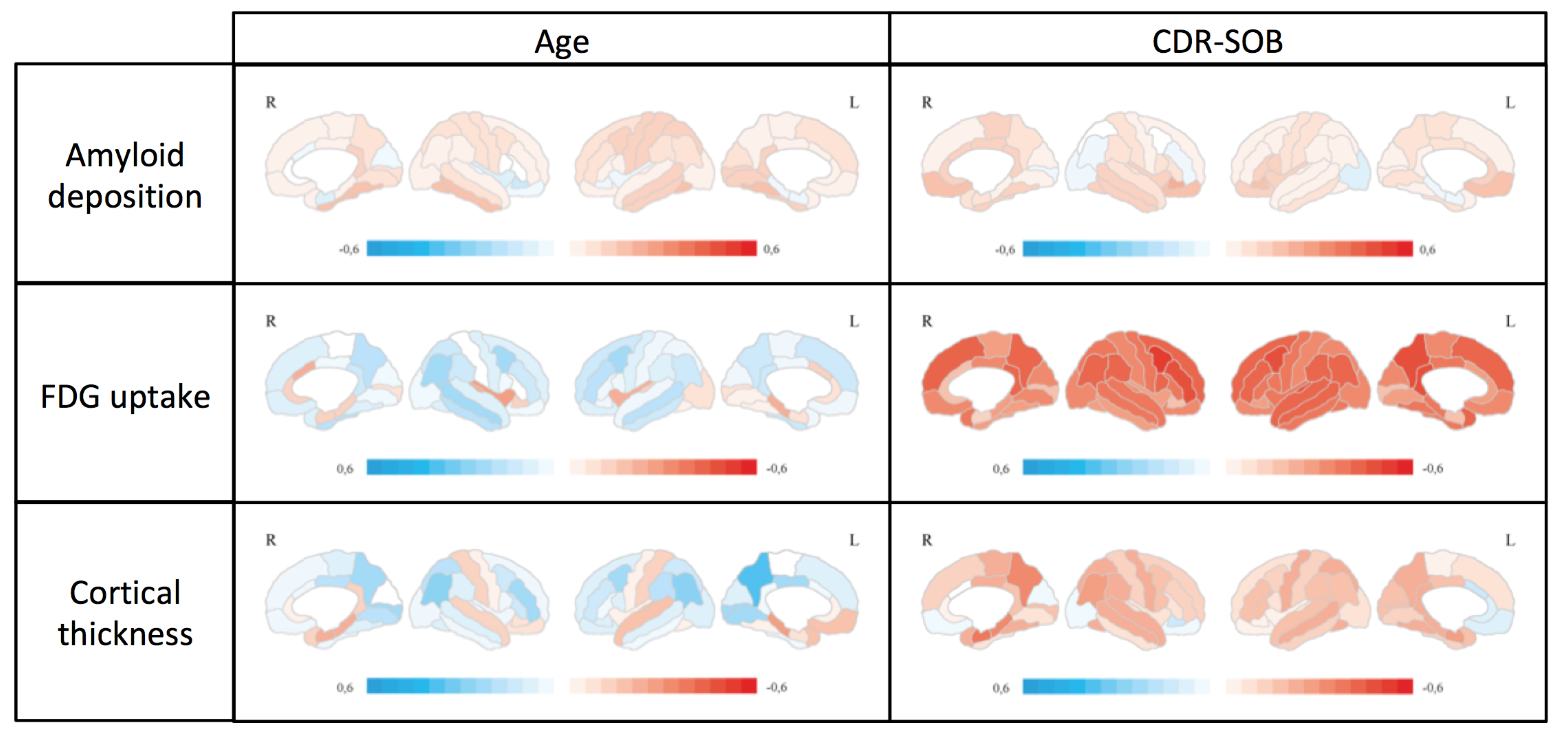


Figure S1: Correlation analyses between age and disease severity (CDR-SOB) and ROI-based imaging biomarkers in the study cohort: Amyloid deposition based on [^11^C] PiB-PET (upper row), FDG-uptake based on [18F]-fluorodeoxyglucose (middle row), and cortical thickness based on structural MRI (lower row). Medial and lateral projections of the right and left hemisphere are shown. Color bars indicate the strength of correlations and orientations were chosen according to Figures 1-3. Maximum Pearson‘s r is set at -0.6 and 0.6, respectively.

Abbreviations: CDR-SOB = Clinical Dementia Rating scale – Sum of boxes; FDG = Fluorodeoxyglucose; MRI = Magnetic Resonance Imaging; PET = Positron emission tomography; PiB = Pittsburgh Compund B; ROI = region of interest.
